# 

**Published:** 1903-05

**Authors:** 


### PERSONALS.

That veteran of the profession, Dr. Wm. Dougherty, of Baltimore, Md., received the well-deserved honor of the Presidency of the A. V. C. Alumni Association at their annual gathering in New York City on April 3, 1903.

Veterinarians Otto G. Noack, of Reading; A. H. Dorney, of Allentown; B. M. Underhill, of Media; and W. L. Rhoads, of Lansdowne, were active factors as delegates from Road Drivers' Associations in the formation at Philadelphia in April of a State organization.

Dr. Leonard Pearson, State Veterinarian of Pennsylvania, addressed the Devon Cattle Breeders' Association at West Chester early in the New Year.

Dr. W. N. Armstrong, graduate of Ontario Veterinary College, Republican candidate for trustee at Concord, Mich., was elected.

Dr. Anderson Crowforth, of Lockport, N. Y., fills the rôle of Worshipful Master of Niagara Lodge, No. 375, F. and A. M.

Dr. W. Horace Hoskins, of Philadelphia, Pa., was one of the guests of the Young Men's Democratic Association at their Jackson Day banquet at the Aldine Hotel.

Dr. Joseph W. Parker, of the Bureau of Animal Industry, has been transferred to Stock Yards Station, Kansas City, Kansas.

Dr. Fred Stehle, graduate of the Veterinary Department of the University of Pennsylvania, has opened an office in one of the palatial boarding stables of Philadelphia.

Secretary Dalrymple, of the Louisiana State Agricultural Society and Louisiana Stock Breeder's Association, announces the annual joint convention of these bodies at Alexandria, La., on April, 22, 23, and 24, 1903.

Leonard Pearson Warner, of Philadelphia, a nephew of Dr. Leonard Pearson, died in the latter part of March, from diphtheria.

Dr. Charles E. Clayton is one of Gotham's veterinarians who abhors Sunday and holiday work. He devotes these periods to a better acquaintance with his growing family.

Dr. D. H. Udall, of St. Johnsbury, Vt., is associated with his brother in a large livery and boarding stable.

Editor Bell, of the *American Veterinary Review*, spent more than a week in bed during the latter part of March wrestling with a painful attack of the grip.

Dr. D. J. Dixon, of Hoboken, N. J., will early retire from the profession to his farm, where he hopes to spend the balance of his days among pastoral scenes and environments.

Dr. J. F. Winchester and L. H. Howard, of Massachusetts, represented the Bay State at the reunion of the A. V. C. boys in New York in April.

